# ^1^H NMR Metabolomics Reveals Association of High Expression of Inositol 1, 4, 5 Trisphosphate Receptor and Metabolites in Breast Cancer Patients

**DOI:** 10.1371/journal.pone.0169330

**Published:** 2017-01-10

**Authors:** Aru Singh, Raj Kumar Sharma, Megha Chagtoo, Gaurav Agarwal, Nelson George, Neeraj Sinha, Madan M. Godbole

**Affiliations:** 1 Department of Molecular Medicine and Biotechnology, Sanjay Gandhi Postgraduate Institute of Medical Sciences, Raebareli Road, Lucknow, India; 2 Centre of Biomedical Research, Raebareli Road, Lucknow, India; 3 Department of Endocrine Surgery, Sanjay Gandhi Postgraduate Institute of Medical Sciences, Raebareli Road, Lucknow, India; University of Nebraska Medical Center, UNITED STATES

## Abstract

^1^H NMR is used to detect alterations in metabolites and their linkage to metabolic processes in a number of pathological conditions including breast cancer. Inositol 1, 4, 5 trisphosphate (IP_3_R) receptor is an intracellular calcium channel known to regulate metabolism and cellular bioenergetics. Its expression is up regulated in a number of cancers. However, its linkage to metabolism in disease conditions has not been evaluated. This study was designed to determine the association if any, of these metabolites with altered expression of IP3R in breast cancer. We used ^1^H NMR to identify metabolites in the serum of breast cancer patients (n = 27) and performed Real-time Polymerase Chain Reaction analysis for quantifying the expression of IP_3_R type 3 and type 2 in tissues from breast cancer patients (n = 40). Principal Component Analysis (PCA) and Partial Least Square-Discriminant Analysis (PLS-DA) clearly distinguished patients with high/low IP_3_R expression from healthy subjects. The present study revealed high expression of IP_3_R type 2 and type 3 in human breast tumor tissue compared to adjacent non-tumorous tissue. Moreover, patients with ≥ 2-fold increase in IP_3_R (high IP_3_R group) had significantly higher concentration of metabolic intermediates compared to those with < 2-fold increase in IP_3_R (low IP_3_R group). We observed an increase in lipoprotein content and the levels of metabolites like lactate, lysine and alanine and a decrease in the levels of pyruvate and glucose in serum of high IP_3_R group patients when compared to those in healthy subjects. Receiver operating characteristic (ROC) curve analysis was performed to show the clinical utility of metabolites. In addition to the human studies, functional relevance of IP_3_Rs in causing metabolic disruption was observed in MCF-7 and MDA MB-231 cells. Results from our studies bring forth the importance of metabolic (or metabolomics) profiling of serum by ^1^H NMR in conjunction with tissue expression studies for characterizing breast cancer patients. The results from this study provide new insights into relationship of breast cancer metabolites with IP_3_R.

## Introduction

Inositol 1,4,5-trisphosphate receptors (IP_3_Rs) are calcium (Ca^2+^) channels that regulate autophagy and metabolism [1].Three different tissue-specific isoforms of IP_3_Rs, namely IP_3_R type 1 (IP_3_R1), IP_3_R type 2 (IP_3_R2) and IP_3_R type 3 (IP_3_R3) have been reported so far [2].These receptors regulate the transfer of Ca^2+^ from endoplasmic reticulum (ER) to mitochondria via a mitochondrial membrane transport protein, which in turn regulates cellular bioenergetics [1]. Altered IP_3_R activity and/or the remodeling of IP_3_R expression profiles may be exploited by cancer cells to promote growth and drug resistance. This becomes important since altered expression of IP_3_Rs have been reported in various cancer types [3, 4]. One of the key areas that can be targeted for potential treatment of cancer is the regulation of metabolism through IP_3_Rs.

IP_3_Rs regulate cell fate by interacting with a number of proteins involved in apoptotic as well as anti-apoptotic pathways [2]. Under normal physiological conditions, IP_3_Rs lead to basal autophagy together with balanced mitochondrial bioenergetics. In contrast, IP_3_Rs in cancer cells cause Ca^2+^ overloading leading to aggressive phenotype depicted as cell survival during therapy [5]. Deregulation of IP_3_Rs play an important role in tumor growth, aggressiveness and drug resistance via modulation of different signaling pathways such as autophagy and energy metabolism [5, 6]. A number of studies reveal altered expression of Ca^2+^ channels and pumps in many human cancers, including breast, ovarian, glioma, liver, pancreatic, prostate, melanoma, colon, lung, bladder, thyroid, and oral cancer [7–11].

NMR Spectroscopy is one of the most widely used techniques in metabolomics studies. NMR-based metabolomics approach using various body fluids and tissue specimens has been applied for diagnosis and pathophysiological studies of various diseases such as coronary heart diseases [12], liver grafts [13], inborn metabolic disorders [14], lung injury [15, 16], hemorrhagic shock [17], liver abscess [18], and many cancers including breast, lung, prostrate, bladder and gall bladder cancers [19–23]. Being non-invasive, blood serum related studies have more potential than using other body fluids, and several studies have so far been reported using serum or plasma [17, 19, 20, 22]. There are many studies which have reported proteomic analyses being used in prognosis of breast cancer. Regular analysis tools like histochemistry and other techniques like MALDI-TOF-MS and SELDI–TOF–MS have been used for studying various breast cancer biomarkers [24–26]. Few studies have been carried out for the metabolic profiling of serum in breast cancer patients using clinical test approach [22, 27].

In the present study, we used metabolomics to differentially identify various metabolites in serum of breast cancer patients with high and low expression of IP_3_R as compared to the ones in serum of healthy controls. Principle Component analysis (PCA) and Partial Least Square- Discriminant Analysis (PLS-DA) were applied to analyze the ^1^H NMR spectra of metabolites in serum samples. The differential metabolites and their pathways along with their ROC analyses are presented.

## Material and Methods

### Collection of Tissues

Blood and tissue specimens were obtained from patients undergoing surgery at Department of Endocrine Surgery, Sanjay Gandhi Post Graduate Institute of Medical Sciences, Lucknow, India. Blood was also obtained from healthy volunteers for use in metabolomics studies. Approval from the Institute's Ethics Committee was taken prior to collection of samples from participants in the study. Written informed consent was obtained from the participants on the format approved by the ethics committee. The study recruited 40 breast cancer patients from whom tissue samples were obtained post-operatively. Out of these subjects only 27 blood samples were available for analysis. Tissues were collected from the tumor zone (tissue within the tumor boundary) and the normal zone (distal normal tissue at least 10 mm from the outer tumor boundary). A fraction of each tissue was fixed in formalin and embedded in paraffin for routine histopathological analysis. The remaining tissue samples were frozen in liquid nitrogen and then stored at -80°C for RNA analysis. The study also recruited 15 age matched healthy controls who volunteered to provide blood sample for the study. Serum samples obtained after centrifugation (at 3000 rpm, 4°C for 10 min) of the blood samples were stored at -80°C for subsequent NMR experiments. Details of patients are given in (S1 Table).

### Real-Time PCR Analysis

Total RNA was isolated from stored tissues using Tri Reagent ^TM^ (Life Technologies, USA) and reverse transcribed to synthesize cDNA using SuperScript^TM^ VILO cDNA Synthesis Kit (Life Technologies, USA) according to the manufacturer's instructions. Real-time PCR was performed using a SYBR mix (Applied Biosystems, USA) as per the manufacturer's instructions. Fold changes in gene expression were calculated using the 2−^ΔΔCT^ method. Specific primers of the genes used for PCR are listed below:

(IP_3_R1): F5'-AACCGCTACTCTGCCCAAAA-3',

R5'-AGTTTGTTGAGTAGCACTGCGTCT-3'

(IP_3_R2): F5'-GCGATCTGCACATCTATGCTG-3',

R5'-AAGTATTAATGTAGGCCCAAGACCTATT-3'

(IP_3_R3): F5'-GGGCTCTCGGTGCCTGA-3',

R5'-GGAGGGCTTGCGGAGAA-3'

(GAPDH): F5′-AGGGCTGCTTTTAACTCTGGT-3',

R5′-CCCCACTTGATTTTGGAGGGA-3′

### Immunohistochemistry

Tissue sections of 3 micron were cut on poly-L-Lysine coated slides. After de paraffinization in xylene and hydration by gradient alcohol series, antigen retrieval was done by heat treatment in citrate buffer (10 mM, pH 6.0). The sections were incubated in 10% NSS (normal sheep serum) for 45 minute to block non-specific binding and further incubated with antibodies against IP_3_R2 or IP_3_R3 (1:200) in 0.1% PBST (Phosphate buffer saline Triton X100) overnight at 4°C. Sections were stained using Quick Universal ABC kit (Vector, USA) followed by peroxidase staining reaction with DAB/H_2_O_2_ as chromogen. The stained sections were observed under bright field light microscope (Nikon Eclipse 80i; Nikon Instech Co. Ltd., Kanagawa, Japan).

### NMR Studies

Serum samples were thawed just before acquiring NMR spectra and 400 μl of each sample was used for the NMR experiment. A co-axial insert containing TSP (sodium salt of trimethylsilyl-2, 2, 3, 3-tetradeuteropropionic acid) was used for deuterium lock as well as for external standard reference. NMR spectra were recorded at Bruker Biospin Avance III 800 MHz NMR (Bruker GmBH, Germany) spectrometer operating at proton frequency of 800.21 MHz and equipped with CryoProbe. Carr-Purcell-Meiboom-Gill (CPMG) pulse sequence was used for acquiring 1D ^1^H NMR spectra. It eliminates the broad resonances arising from the macromolecules (proteins and lipids). CPMG experiment provides spectra with smooth baseline which facilitates multivariate analyses. NMR spectra of patient and healthy control samples were recorded with constant parameters to ensure accuracy of the results. All spectra were recorded with 64 k time domain data points, 20.02 ppm spectral width, 128 scans, relaxation delay of 5 s, and constant receiver gain value of 80.6 and 400 ms echo time. TOPSPIN software (version 2.1) was used for phase and base line correction of spectra. Two-dimensional homo-nuclear (^1^H-^1^H COSY and^1^H-^1^HTOCSY) and hetero-nuclear (^1^H-^13^C HSQC) data were also acquired to confirm the resonance assignment. Assignment of metabolites was also confirmed from a standard database [21].

### Cell Culture

The human cancer cell line, MCF-7 cells (Invasive ductal carcinoma, Estrogen/Progesterone receptor positive cell line), MDA MB-231 (Adenocarcinoma, Estrogen/Progesterone/Her2+ receptor negative or triple cell line) and MCF 10A (non tumorous mammary cell line) were procured from National Centre for Cell Sciences (NCCS), Pune, India and American Tissue & Cell Culture (ATCC) were used in the present study. MCF-7 cells were cultured in DMEM-F12 (Life Technologies, USA) and MDA MB-231 in DMEM supplemented with 10% fetal bovine serum. MCF 10 A cells were cultured in DMEM/F12 media supplemented with supplemented with 5% horse serum (Life Technologies, USA) along with cholera toxin (100ng/ml), Epidermal growth factor (EGF 20ng/ml), hydrocortisone (500ng/ml), insulin (10μg/ml) (Sigma,USA) and 1% penicillin, streptomycin antibiotic mixture (Life Technologies, USA). Cells were incubated at 37°C and 5% CO_2_ in specified media.

### Cell Treatment

MCF-7, MDA MB-231 and MCF 10A cells (2×10^6^) were seeded in 6-well plates and treated with either vehicle (Control) or 25 μM XeC (inhibitor of IP3R mediated calcium release) (Cayman) in calcium free medium (EGTA pretreatment,1mM,was given to chelate extracellular calcium) for 48 hours. MCF-7, MDA MB-231 and MCF 10A cells were transfected using Lipofectamine 2000 transfect reagent (Invitrogen, USA) as per the manufacturer's protocol. Briefly, 2×10^6^cells were transfected with 4μg siRNA directed against the human IP3R2 or IP3R3 mRNA sequence (ON-TARGET plus, Dharmacon, USA) or control siRNA (siGENOME non-targeting siRNA; Dharmacon, USA) for 72 hours and further analyses were performed.

### Measurement of Glucose Uptake

Percent glucose uptake was determined in MCF-7, MDA MB-231 and MCF 10A cells plated in 96-well plates and treated with vehicle alone or 25 μM XeC for 24 hours, or with siC (non-targeted siRNA) or siIP_3_R2 or siIP3R3 for 72 hours following respective protocols provided by the cell based assay kit manufacturer (Cayman, USA). Glucose uptake was analyzed using {2-[*N*-(7-Nitrobenz-2-oxa-1, 3-diazol-4-yl] Amino}-2-Deoxyglucose (NBDG), a fluorescently labeled deoxy glucose analogue as a probe for detection of glucose taken up by cultured cells.

### RT2 Profiler PCR Array

MCF-7 cells were plated in 6-well plate, treated with vehicle alone or XeC for 24 hours. Total RNA was isolated from the treated- and untreated- MCF-7 cells by a single step method using TRIZOL^®^ reagent (MRC, USA). RNA was converted into first-strand cDNA using the RT2 First Strand Kit (Qiagen, Germany). Further, the cDNA was mixed with an appropriate RT2 SYBR Green Mastermix (Qiagen, Germany). This mixture was aliquoted into the wells of the RT2Profiler PCR Array. Real time PCR was performed and relative expression was determined using 2-ΔΔCT method. The detailed protocol as per the manufacturer’s instructions (RT2 Profiler assay, Qiagen, Germany) was followed.

### Statistical Analysis

Multivariate statistical analysis was performed on 1D CPMG data collected from serum samples of patients and healthy control subjects. Further data reductions were applied for statistical analysis. 0.03 ppm bin file were generated using Amix software and exported to the ‘Unscrambler X’ Software package (Version10.0.1, Camo USA, Norway). Spectral alignment was checked prior to statistical analysis. Supervised Principal Component Analysis (PCA) and unsupervised Partial Least Square Discriminant Analysis (PLS-DA) was done using bin file. Model validation was checked by employing a 7-fold internal cross validation procedure which gives the model validity in the form of explained variance results (R^2^) and predictive capability results (Q^2^).

Quantitative analysis was performed by taking relative integral areas. Integral values of these metabolites were compared for serum samples from both the groups. Student t-test was performed to test the significance of metabolite variations within groups. ROC analysis was performed for area under curve (AUC) of metabolites to predict the diagnostic significance of metabolites using Graph Pad Prism software. Metaboanalyst 3.0 was used for pathway analysis.

All cell line experiments were repeated at least three times. The data were expressed as mean ±S.E. (Standard Error) from three independent experiments. Statistical analysis was performed by using ANOVA followed by post hoc Newman-Keuls test or Student’s t test (two-tailed) while comparing multiple or two groups. The criterion for statistical significance was taken as p< 0.05.

## Results

### Dysregulated Inositol 1, 4, 5-Trisphosphate Receptors (IP_3_Rs) in Breast Cancer Tissue

**Fig 1:**
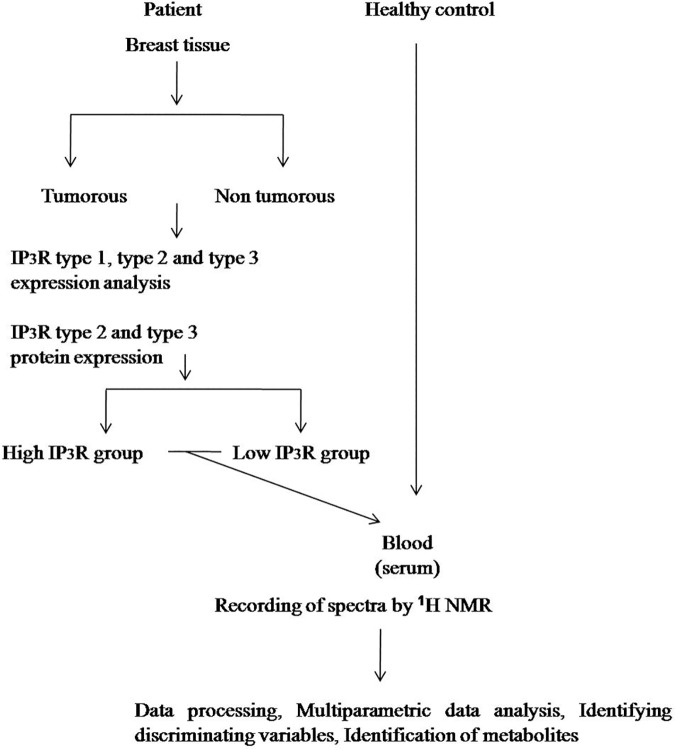
An overview of the workflow performed for the serum metabolic profiling of breast cancer patients using ^1^H NMR spectroscopy.

**Fig 2:**
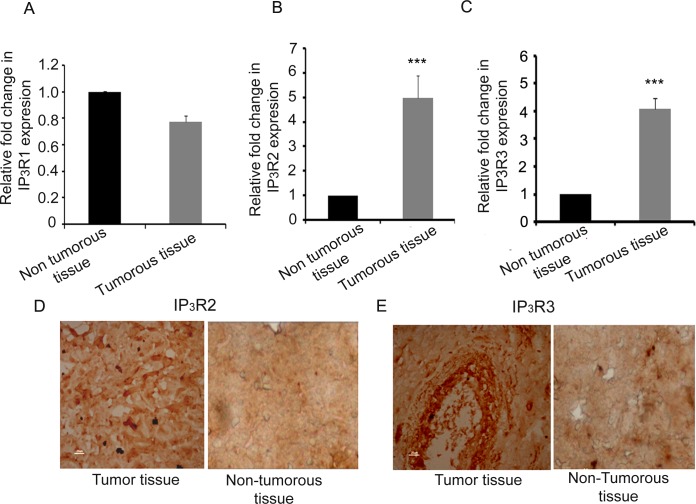
Analysis of expression of Inositol 1, 4, 5-trisphosphate receptors type 2 (IP_3_R2) and type 3 (IP_3_R3) in tumor tissue of breast cancer patients. Relative mRNA expression of (A) IP_3_R1, (B) IP_3_R2 (p<0.001) and (C) IP_3_R3 (p<0.001) from tumoral and extra-tumoral tissues of breast cancer patients was analyzed using Real -time PCR. Analysis of tumoral and extra-tumoral tissues was done by incubating the tissue sections with antibodies against (D) IP_3_R2 and (E) IP_3_R3, and detected using DAB staining.

The complete scheme for the analysis of test results of patients employed in this study is shown in (Fig 1). Expression levels of IP_3_R2 and IP_3_R3 mRNA transcripts were investigated in tissues obtained from breast cancer patients using quantitative real-time PCR. IP_3_R1 was not significantly different in breast tumor tissue from the adjacent non-tumorous tissue with average increase in tumor volume ~0.77-fold relative to non-tumorous tissue (S.E.±0.28, Fig 2A). Average fold change of IP_3_R2 and IP_3_R3 transcript levels were significantly higher (~4- fold) in tumor tissue compared to the adjacent non-tumorous region from the same patient (p<0.001, n = 30, Fig 2B and 2C). Further, immunohistochemistry revealed intense staining of IP_3_R2 and IP_3_R3 in the tumor region as compared to the adjacent non-tumorous region (Fig 2D and 2E).

### Metabolic Profile of Serum in Breast Cancer by ^1^H NMR

**Fig 3:**
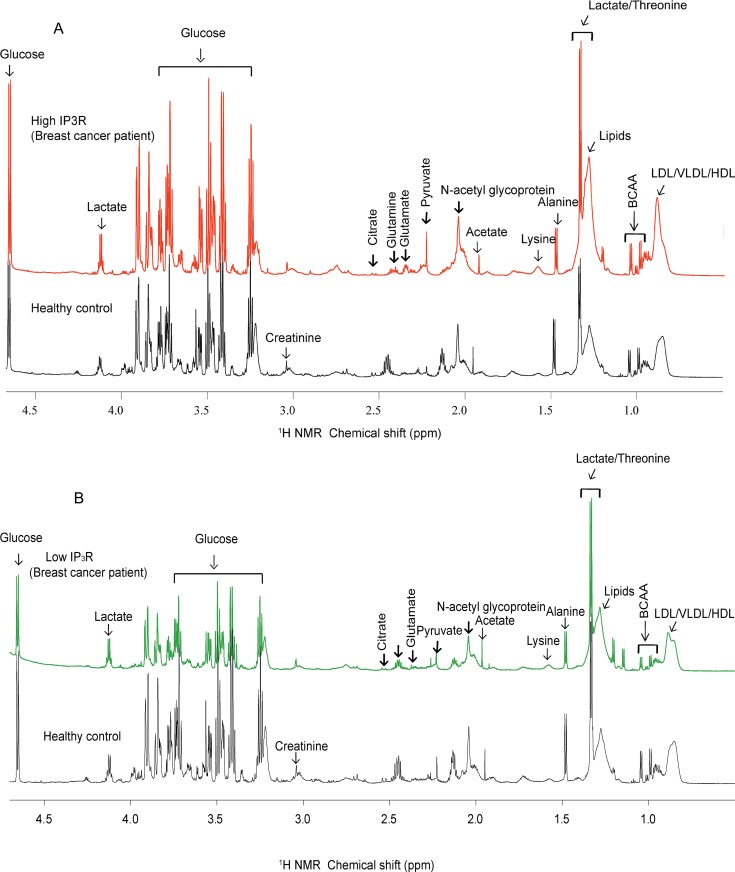
**Stack plot of representative** ^**1**^**H NMR CPMG spectra of serum from A) healthy control and patient with high tissue expression of IP**_**3**_**R, B) healthy control and patient with low tissue expression of IP**_**3**_**R.** All the spectra were plotted at same vertical scale for quantitative comparison.

^1^H CPMG spectra depicting comparison between healthy subjects and patients (High IP_3_R and Low IP_3_R) is reported in (Fig 3A and 3B). Spectrum of serum from patients and healthy subjects shows the presence of metabolites including amino acids like alanine, lysine, glutamine, glutamate, other branched chain amino acids, metabolites like lactate, N-acetyl glycoproteins (NAG), glucose, lipids, creatinine, acetate, pyruvate, citrate and lipoproteins (HDL, LDL and VLDL). There were no marked qualitative and quantitative differences between spectra from healthy subjects and low IP_3_R patients. In contrast, quantitatively spectra from healthy control and high IP_3_R patient groups differed significantly with spectral quality remaining same. The metabolites making significant contribution for group separation were glucose, lactate, lysine, alanine, glutamate, NAG, pyruvate and lipid content. These differences were further confirmed by applying multivariate analyses like PCA and PLS-DA.

### Multivariate Analysis of ^1^H NMR Spectra of Serum: Discrimination Between Healthy Control and Patient (high IP_3_R and low IP_3_R) Groups

**Fig 4:**
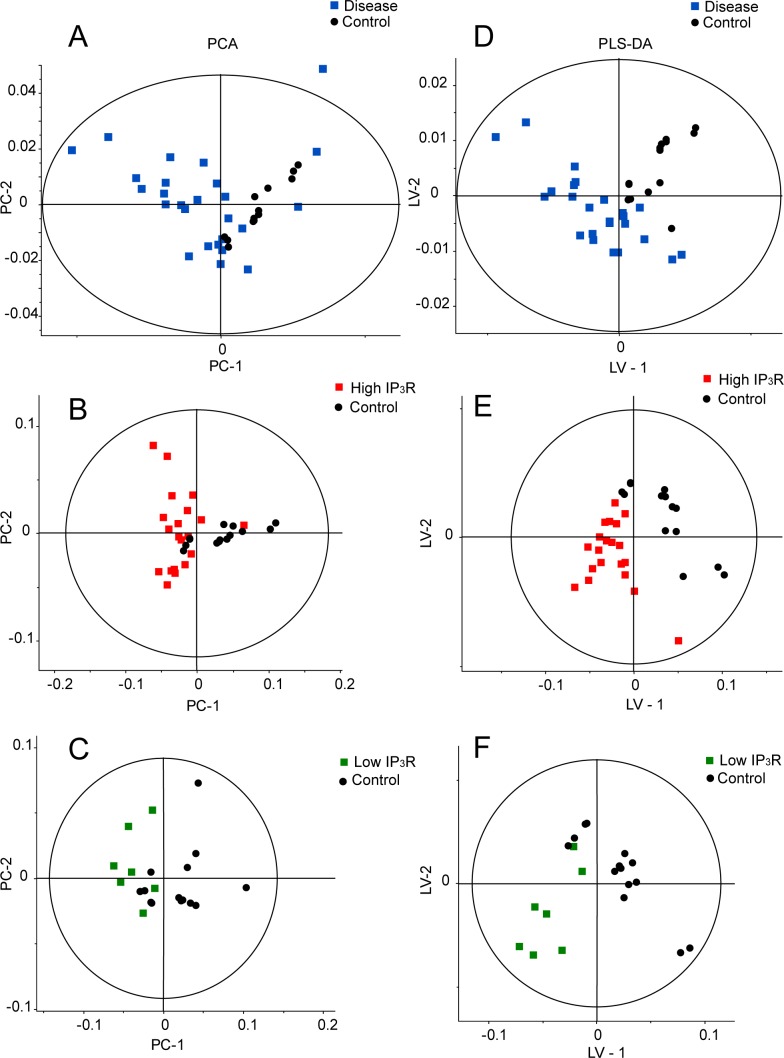
Score Plot Generated From PCA And PLS-DA Analysis Of NMR Spectra Of Serum From Healthy Control And Patient Groups. (A) PCA and (D) PLS-DA score plot generated for healthy control vs. patient group, (B) PCA and (E) PLS-DA score plot generated for healthy control and high IP_3_R group, (C) PCA and (F) PLS-DA score plot generated for healthy control and low IP_3_R group.

The NMR spectra recorded for serum samples were subjected to multivariate data analysis to unravel changes in the serum metabolic profiles and identify the potentially altered metabolic pathways in patients with differentially expressing IP_3_R gene with respect to healthy controls. The bias in this study is negated with the help of multivariate analysis which itself normalizes the data and separates the groups after normalizing the bias in the data. PCA score plots were generated initially for an overview of the dataset. PCA scores for all groups were obtained as shown in (Fig 4A). PCA analysis was performed with integral data for healthy control versus patients. The scattered score plot of PCA revealed that healthy controls were well clustered and distinguishable from patient groups (healthy control and high/low IP_3_R) (Fig 4B and 4C). Moreover, the outcome of PCA showed clustering of samples according to the disease milieu.

**Fig 5:**
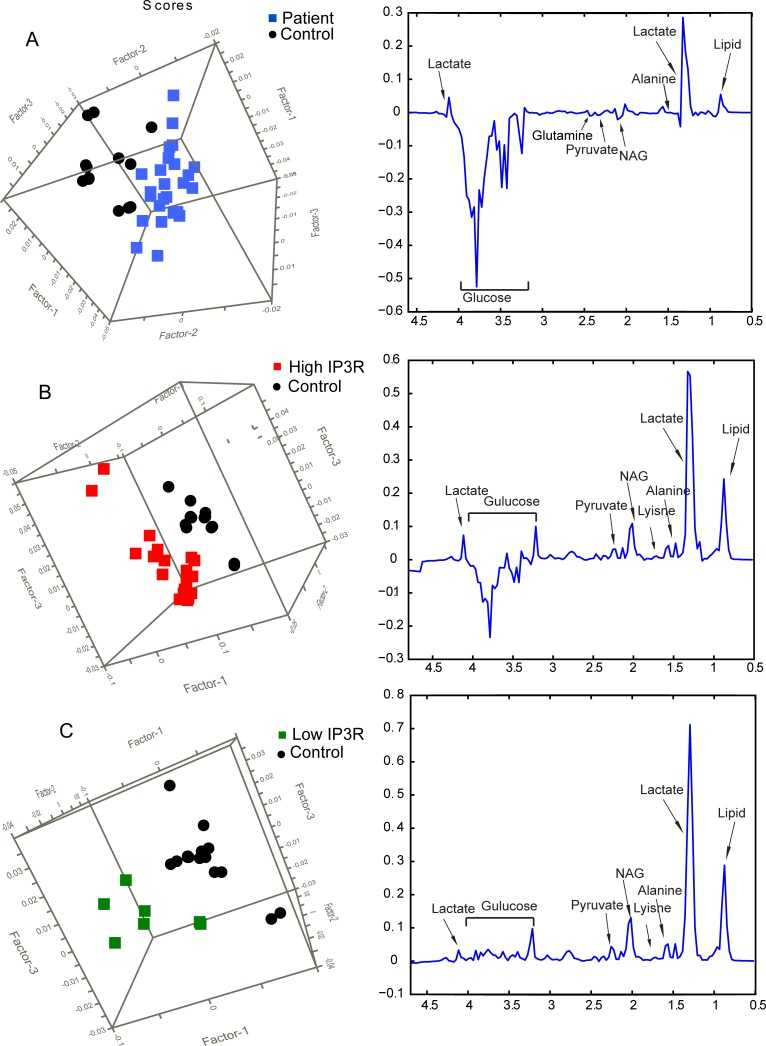
Score plot and corresponding loading plot generated from PLS-DA analysis between (A) healthy control and patient group, (B) healthy control and high IP_3_R group, (C) healthy control and low IP_3_R group.

To obtain a more specific statistical analysis, PLS-DA was employed on all the serum samples, and the results showed a clear separation and a clustered pattern among the various groups in the PLS-DA score plot as shown in (Fig 4D). The goodness of fit and predictability for the PLS-DA models for healthy control versus patient group was reflected in the values of R^2^X = 0.85, R^2^Y = 0.77 and Q^2^ = 0.85. A pair wise PLS-DA analysis (R^2^X = 0.97, R^2^Y = 0.85 and Q^2^ = 0.92) between groups was also performed for healthy control versus high IP_3_R group (Fig 4E) and healthy control versus low IP_3_R group (Fig 4F). The analysis demonstrated well-segregated and clustered form of PLS-DA score plots. We further analyzed the metabolites which made the groups different as depicted in the loading plot (Fig 5A–5C). The discriminating metabolites between the groups were identified as lipids, lactate, glutamate, glucose, lysine, alanine, pyruvate, and NAG.

### Quantitative Analysis of Metabolites

**Fig 6:**
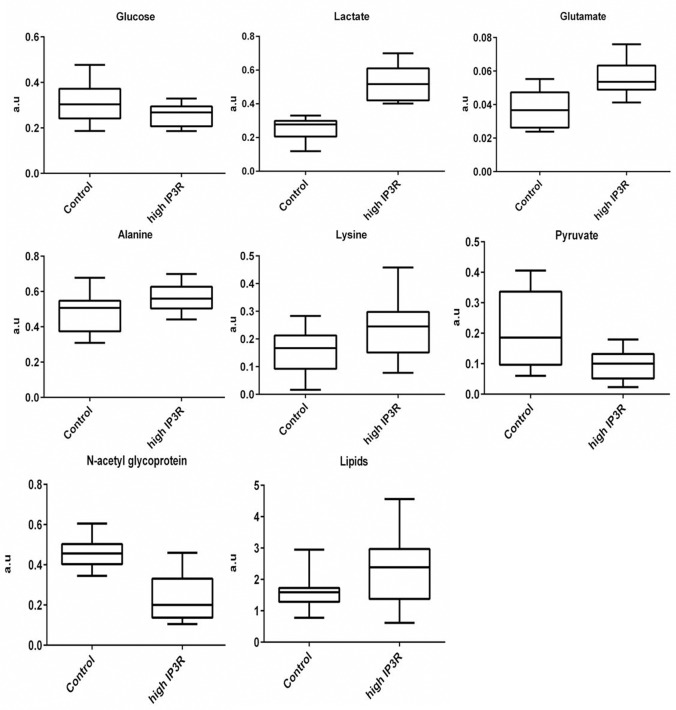
Representative box-cum-whisker plots for significant metabolites of healthy control vs high IP_3_R patient group (with p>0.05). Relative integral areas are calculated from CPMG spectra and plotted as a.u.

Pair wise PLS-DA comparison of healthy control with patients with high tissue expression of IP_3_R revealed several metabolites as discriminatory metabolites. The list of these discriminatory metabolites is given in (Table 1) wherein relative integral areas of these metabolites were calculated and compared between healthy controls and patient groups by use of statistical analysis (p<0.05) as reported in (Fig 6).

**Table 1:** Metabolites contributing to the difference between healthy control and high IP_3_R patient group.

Metabolites	Chemical shift (multiplicity)	Trend		AUC^a^ of ROC	p value
		High IP3R	Low IP3R		
Glucose	3–4 ppm	↓	-	0.89	<0.001
Lactate	4.10 (q)	↑	-	1.00	<0.001
Glutamate	2.42 (m)	↑	-	0.85	<0.001
Lysine	1.69 (m)	↑	-	0.71	<0.05
Alanine	1.46 (d)	↑	-	0.70	<0.001
Pyruvate	2.2 (s)	↓	-	0.73	<0.01
NAG	2.1 (m)	↓	-	0.93	<0.001
Lipids	1.3 (m)	↑	-	0.70	<0.05

^a^AUC (Area under curve) of ROC. Integral areas of normalized spectral regions were calculated and significance was obtained using student t test. Significant differences shown with different symbols, represent significant increase in patient with high expression of IP_3_R as compared to healthy control; (↑) represents significant decrease compared to control; (↓) represents no significant difference; m = multiplet; q = quartet; d = doublet and s = singlet.

Resonances arising from lipids were significantly higher in patients with high tissue expression of IP_3_R as compared to healthy control. Patients with low tissue expression of IP_3_R showed no significant difference compared to healthy control. Among the branched chain amino acids (valine/leucine/isoleucine) there were no significant differences between healthy control and patient groups. There was no change in aromatic amino acids in any of the patient groups as compared to healthy control. A high energy molecule like glucose was found to be significantly decreased in patients with high expression of IP_3_R when compared to healthy control. Lactate was found to be significantly higher in patients with high tissue expression of IP_3_R when compared to patients with low tissue expression of IP_3_R and healthy control. Lactate concentration was higher in patients with low tissue expression of IP_3_R as compared to healthy controls. The high IP_3_R group had low levels of pyruvate as compared to healthy control, while there was no significant difference observed in case of low IP_3_R group for the resonance of pyruvate when compared to healthy control. Amino acids like alanine and lysine were significantly increased in all patient groups compared to healthy control, but only patients with high IP_3_R showed significant increase. Glutamate was found to be elevated in patients with high IP_3_R while there was no significant difference between low IP_3_R group and healthy control. NAG levels were found to be reduced in both the patient groups. The decrease in NAG levels was greater in case of patients with high IP_3_R than with low IP_3_R or healthy control.

### Inhibiting or Silencing Inositol 1,4,5-trisphosphate Receptors (IP_3_Rs) Compromises Cellular Bioenergetics in Breast Cancer Cells

**Fig 7:**
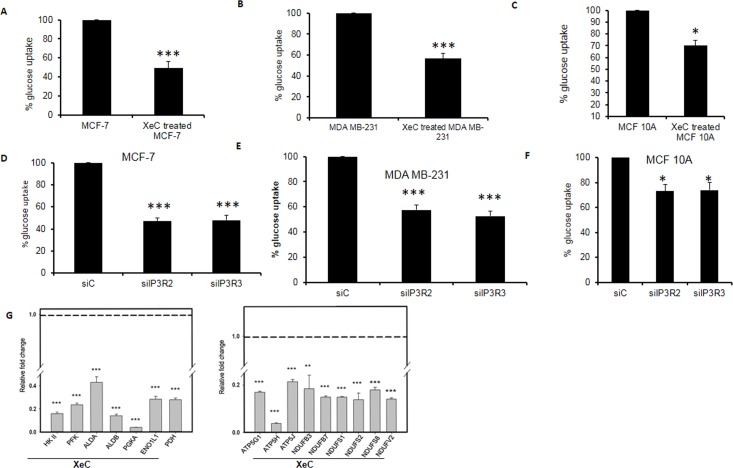
Inhibition of IP_3_R in MCF -7 breast cancer cells effects metabolism. Glucose uptake in MCF-7 cells was analyzed using NBDG a fluorescently labeled deoxy glucose analogue as a probe for detection of glucose taken up by cultured cells. Quantitative estimation of glucose uptake, using a cell based assay kit, was performed as per instructions provided by the manufacturer (Cayman, USA). Cells were plated in 96-well plates and treated with 25 μM XeC for 24 hours or with siC (non-targeted siRNA) or siIP_3_R2 or siIP_3_R3 (72 hours,Fig7A and 7D) Representative graph in MCF-7 cells. (Fig 7B and 7E) Representative graph showing percentage of glucose uptake as estimated using cell based assay in MDA MB-231 cells. (Fig7C and 7F) Representative graph showing percentage of glucose uptake as estimated using cell based assay in MCF 10A cells. RNA was extracted from treated and untreated cells and cDNA was prepared (Fig 7G) RT profiler PCR array for glucose as well as mitochondrial metabolism genes was performed using cDNA prepared from mRNA of MCF-7 cells. Data represent mean ±SEM. *p< 0.05, ***p< 0.001 compared to vehicle.

In order to demonstrate the functional relationship between IP_3_R and metabolites, we investigated the effect of pharmacological inhibition and siRNA silencing of IP_3_R in cancerous cell lines in 2 separate experiments. A significant decrease was observed in glucose uptake by blocking/silencing the IP_3_R receptor in MCF-7, MDA MB-231 and MCF 10 cells (Fig 7A–7F). However the reduction of glucose uptake was more pronounced in MCF 7 and MDMBA-231 cells as compared to MCF 1OA cells. Further, we performed RT^2^profiler assay which revealed a significant fold reduction in the expression levels of key glycolytic and mitochondrial pathway genes in MCF-7 cells treated with IP_3_R inhibitor compared to vehicle treated cells (Fig 7G p<0.001. These results showed that *in vitro* inhibition of IP_3_R result in compromised bioenergetics, both in terms of glucose and mitochondrial metabolism.

## Discussion

Tumorigenesis is the process of cancer formation where by normal cells are transformed into cancerous cells. This process is associated with a series of metabolic events that fuel cells to multiply or become aggressive [28]. IP_3_R channels are calcium channels which play a crucial role in regulating metabolism in cells [1]. In the present study, we targeted patients with altered expression of IP_3_R. Elucidation of metabolic changes involved in the pathogenicity with respect to IP_3_R can be helpful for exploring novel biomarkers for the diagnosis and surveillance of the disease. This study brings forth several important findings of NMR-based metabolomics of human serum samples from breast cancer patients. We report altered expression of IP_3_R2 and IP_3_R3 while IP_3_R1 remain unaltered in tumor tissue compared to adjacent non-tumorous tissues from breast cancer patients. As IP_3_R is also known to regulate autophagy and metabolism [1], we also compared serum metabolic profiles among healthy subjects and breast cancer patients with high IP_3_R and low IP_3_R. We determined that the NMR-derived fingerprint of the serum metabolic profile is able to help discriminate among breast cancer patients with high and low IP_3_R expression and healthy controls. Interestingly, PCA and PLS-DA multivariate analysis not only differentiated the groups, but also revealed potential biomarkers from the complex NMR spectra of serum. Absolute quantitation and measurement of important metabolites using integral values established accuracy of these results. Lastly, the ROC curve analysis of vital biomarkers provided the clinical relevance of the essential metabolites. In addition, the *in vitro* results showed that inhibition of IP_3_R resulted in reduced glucose uptake in breast cancer cells -MCF-7 and MDA MB-231.

**Fig 8:**
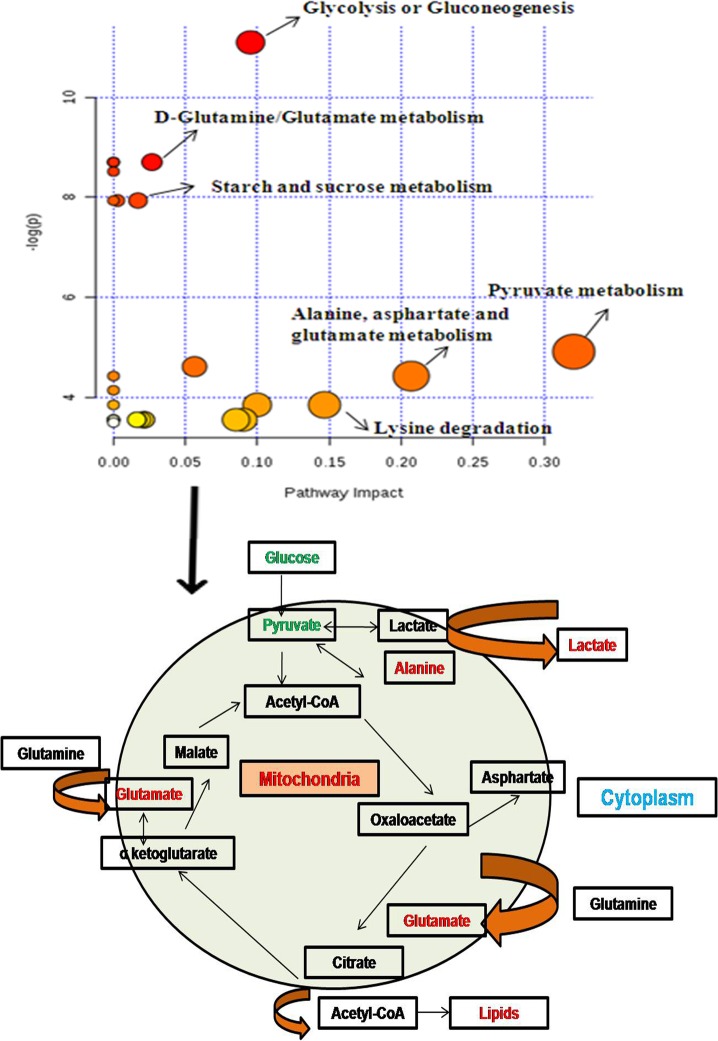
An integrated analysis based on Metaboanalyst software (pathway tool) for a simplified view of contributing pathways. The panel shows a view of metabolism in cancers depicting glycolysis and Krebs cycle which are modified to different processes like lipid and amino acid synthesis to meet the requirement of proliferating cells. (Metabolites depicted with green/red are decreased/increased in the present study).

Dysregulated metabolic pathways in breast cancer patients with high IP_3_R were identified on the basis of the metabolites that exhibited significant change in the NMR spectra of serum samples. A simplified metabolic pathway is demonstrated in Fig 8 based on Metaboanalyst software. The analysis showed alteration in a number of biochemical pathways like glycolysis, pyruvate metabolism, alanine/aspartate/glutamate metabolism and lysine degradation. The analysis was calculated based on the significance value (p<0.05) of the pathway enrichment analysis. Here “Impact” is the pathway impact value calculated from pathway topology analysis and a value equal to or greater than 0.1 was considered significant.

Deregulated expression of IP_3_Rs has been reported in various cancer types [3, 4]. Deregulation of IP_3_Rs aids in growth and aggressiveness of the tumor, and also drug resistance through different signaling pathways such as autophagy and energy metabolism [5, 6]. Through this study, we are the first group to report dysregulated expression of IP_3_R2 and IP_3_R3 while IP_3_R1 remains unaltered in breast cancer tissue compared to their expression in adjacent non-tumorous tissues from the patients. Increased expression of IP_3_R3 has been associated with tumor aggressiveness in other cancer types such as colorectal carcinoma and gastric cancer [3, 4]. Similarly, IP_3_R2 was found to be increased in lymphomas which makes it susceptible to be targeted for its association with Bcl-2 [29]. We also observed that patients with more than 2-fold of IP_3_R (both IP_3_R3 and IP_3_R2) tissue expression also had higher concentration of metabolic intermediates in serum analyzed by ^1^H NMR spectroscopy. It has been demonstrated that blocking/knocking out IP_3_R in MCF-7 breast cancer cells compromises bioenergetics and induces autophagy [1].

A number of studies have shown modified expression of Ca^2+^ channels and pumps in a number of human cancers like breast, ovarian, glioma, liver, pancreatic, prostate, melanoma, colon, lung, bladder, thyroid, and oral cancers [7, 8, 10, 11, 30, 31]. Calcium transfer through these channels, especially IP_3_R is known to regulate metabolism. There are also various online datasets available showing the expression of IP3Rs as well as its link to metabolism. A search of GEO profiles database revealed high expression of IP_3_R3 in breast cancer patients (GEO accession: GDS1250) [32]. Another study in GEO profiles database showed analysis of A549 lung carcinoma and M059K glioblastoma cells treated with dichloroacetate (DCA), an inhibitor of the mitochondrial pyruvate dehydrogenase kinase. The expression of IP_3_R3 was down regulated in this study showing its link to metabolism (GEO accession: GDS2444) [33]. Furthermore, a genome wide expression analysis in HPV16 cervical cancer revealed altered metabolic pathways and increased expression of IP_3_R[34].

It has been demonstrated that ^1^H NMR spectroscopy is a potential tool for identifying metabolic perturbations in a number of pathological conditions using various body fluids (e.g. cerebrospinal fluid, serum, urine, plasma etc.) [35, 36]. Many studies to date have used NMR or MS methods to detect altered metabolic profiles in different types of malignancies due to their capability to analyze a large number of metabolites in a single experiment [22, 37]. In particular, several investigators, by using a metabolomics approach, have focused on establishing breast cancer biomarkers [38–40]. Numerous metabolites, including glucose, lactate, lipids, choline, and amino acids were shown to correlate with breast cancer [40–42]. A majority of these investigations were focused on either breast cancer tumors or cell lines. All these studies utilized NMR methods alone except for a study that utilized a combination of NMR and MS methods [43].

In the present study, role of ^1^H NMR spectroscopy was explored in serum of breast cancer patients with high tissue expression of IP_3_R in comparison to healthy controls after characterizing the patients under different groups based on their IP_3_R expression status. A total of 8 metabolites have been identified in studies associated with breast cancer [27, 41]. These metabolites represent changes in one or the other metabolic pathways involved in cancer cell bioenergetics including amino acid metabolism (glutamic acid, alanine, and lysine), glycolysis or gluconeogenesis (glucose, lactate, and pyruvate) and glutamate metabolism (glutamate). Increased lactate, reduced glucose and pyruvate, as reported in our study, are among the early findings of metabolic changes reported for breast tumors. These changes are associated with high rate of glycolysis in cancer cells. Also, association of a number of amino acids, fatty acids, and organic acids with breast cancer has previously been shown [22, 27]. High levels of glutamate in the present study can be associated with an activation of glutaminolysis through an increased activity of the mitochondrial enzyme glutaminase. Glutamate has been recognized in a few studies as a potential biomarker related to various types of cancers and more recently in breast tissues as a tumor biomarker [44–47]. As shown by our data, the mean concentrations for a number of these metabolites, including alanine and lysine, were found to be increased while that of N-acetyl-glycoprotein was decreased significantly in patients with high expression of IP_3_R (in comparison to healthy control). Alanine is a well-known tumor biomarker [48, 49]. It is required for tumor proliferation and is a product of glycolysis/glutaminolysis. On the other hand, there was no significant change in the concentration of these metabolites in patients with low expression of IP_3_R when compared to healthy control. Most of the intermediates found in our study are of glycolytic pathway in patients with high IP_3_R, thereby revealing greater dependence on glucose metabolism. Our findings highlights the correlation of these metabolites with the expression of IP_3_R in breast cancer patients.

Providing evidence regarding the functional relationship between IP_3_Rs and metabolic disruption albeit of importance cannot be done in patient material. As a rational alternative, we investigated the effect of inhibition of IP_3_R in MCF-7, MDA MB-231, MCF 10A cells using pharmacological inhibitor XeC and siRNA approach. IP_3_Rs are known to regulate cellular metabolism and bioenergetics. Blocking of IP_3_R by XeC as well as siRNA silencing of IP_3_R2 and IP_3_R3 in MCF-7 cells and MDA MB- 231 resulted in significant reduction in a key metabolic process such as % glucose uptake. MCF 10A which is normal cell line was not effected to the extent observed in cancer cell lines (MCF-7 and MDA MB-231) showing the differential effect of IP_3_R blocking / silencing. Further, RT^2^ profiler assay revealed a significant fold reduction in the expression levels of key glycolytic and mitochondrial pathway genes in MCF-7 cells treated with IP_3_Rs blocker compared to vehicle treated cells. Thus, inhibiting IP_3_R affected the glucose and mitochondrial metabolism whose metabolites were utilized as a source of energy with high IP_3_R expression in breast cancer patients.

Through this preliminary study, we have shown an association between IP3R and dysregulated metabolism in breast cancer patients. The relevance of our in vitro findings can be further explored by carrying out similar studies in primary cultures from human tissues as well as animal models.

While metabolic reprogramming is one of the major hallmarks that differentiate cancer from normal cells, unchecked cell proliferation not only includes deregulation of proliferation but also alterations in energy metabolism to supply metabolites and cofactors required for cell growth and division [50]. The modified metabolic schemes depend on glycolytic activity, the Warburg effect, and on elevated glutamine metabolism [51]. While assessment of circulating metabolites may not reflect the tumor metabolism alone, it provides a global picture of the balance between tumor metabolism and the physiological condition of cancer patients. In current study we have presented NMR based metabolomics application to show the correlation between IP3R expression and serum metabolites. The differential metabolites can potentially be targeted for providing treatment options for future.

## Conclusions

In conclusion, our study provides serum metabolic profile of breast cancer tissues with high IP_3_R. Despite the variations among patients, similarities in metabolic alterations observed in the tumor samples resulted in clear group separation from the non-tumor samples. The findings from our study are consistent with the understanding of cancer metabolism. Further mechanistic insights need to be elucidated using *in vitro* studies for better understanding of the targets which are regulated through IP_3_R in cancer metabolism.

## Supporting Information

S1 Table Summary of clinicopathological characteristics of breast cancer patients. Hormone receptors are receptors for estrogen and progesterone; HR-: at least one of the two receptors (estrogen and progesterone) is negative; HR+: both receptors are positive.
(DOCX)
