# Supplementary material for: 1H NMR Metabolomics Reveals Association of High Expression of Inositol 1, 4, 5 Trisphosphate Receptor and Metabolites in Breast Cancer Patients
Source: PLoS One. 2017 Jan 10;12(1):e0169330. doi: 10.1371/journal.pone.0169330 (PMC5225010; doi:10.1371/journal.pone.0169330)

| **Charactertics** | **Healthy control** | **Patients** |
| --- | --- | --- |
| **Number of subjects** | **15** | **40** |
| **Mean age** | **55.2 ± 11.2** | **58.6 ± 12.4** |
| **Breast cancer stage** |  |  |
| **II** | **-** | **15** |
| **III** | **-** | **25** |
| **HR+** | **-** | **29** |
| **HR-** | **-** | **11** |

**^1^H NMR metabolomics reveals association of high expression of Inositol 1,4,5 trisphosphate receptor and metabolites in breast cancer patients**

Aru Singh^1#^, Raj Kumar Sharma^2#^, Gaurav Agarwal^3^, Neeraj Sinha^2^, Madan M Godbole^1*^

**S Table 1.** Summary of clinicopathological characteristics of breast cancer patients. Hormone receptors are receptors for estrogen and progesterone; HR-: at least one of the two receptors (estrogen and progesterone) is negative; HR+: both receptors are positive.

**Supplementary Table 2**. Metabolites contributing to the difference between control and patient groups (healthy control with high IP_3_R group) along with their chemical shift, multiplicity and AUC of ROC. Integral areas of normalized spectral regions were calculated and significance was obtained using student t test. Significance differences are shown with different symbols, ( ) represents significant increase in patient with high expression of IP_3_R as compared to control; ( ) represents significant decrease compared to control; (-) represents no significant difference; m= multiplet; q=quartet; d=doublet and s=singlet.


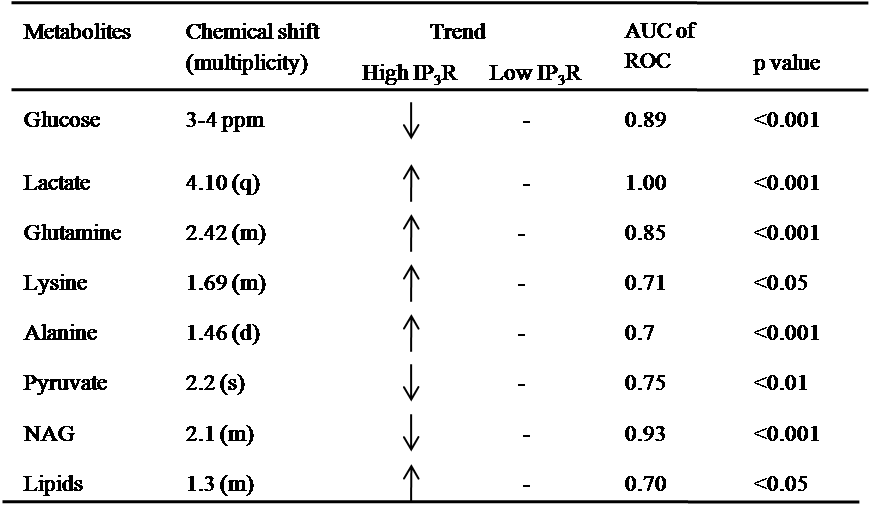

Supplement: S1 Table — Hormone receptors are receptors for estrogen and progesterone; HR-: at least one of the two receptors (estrogen and progesterone) is negative; HR+: both receptors are positive. (DOCX) [file pone.0169330.s001.docx]
